# The Multifaceted Roles of B Cells in the Thymus: From Immune Tolerance to Autoimmunity

**DOI:** 10.3389/fimmu.2021.766698

**Published:** 2021-11-01

**Authors:** Justine Castañeda, Yessia Hidalgo, Daniela Sauma, Mario Rosemblatt, María Rosa Bono, Sarah Núñez

**Affiliations:** ^1^ Departamento de Biología, Facultad de Ciencias, Universidad de Chile, Santiago, Chile; ^2^ Cells for cells-Consorcio Regenero, Universidad de Los Andes, Santiago, Chile; ^3^ Facultad de Medicina y Ciencia, Universidad San Sebastián, Santiago, Chile; ^4^ Fundación Ciencia y Vida, Santiago, Chile

**Keywords:** B cells, thymus, central tolerance, aging, autoimmune disease

## Abstract

The thymus is home to a significant number of resident B cells which possess several unique characteristics regarding their origin, phenotype and function. Evidence shows that they originate both from precursors that mature intrathymically and as the entry of recirculating mature B cells. Under steady-state conditions they exhibit hallmark signatures of activated B cells, undergo immunoglobulin class-switch, and express the Aire transcription factor. These features are imprinted within the thymus and enable B cells to act as specialized antigen-presenting cells in the thymic medulla that contribute negative selection of self-reactive T cells. Though, most studies have focused on B cells located in the medulla, a second contingent of B cells is also present in non-epithelial perivascular spaces of the thymus. This latter group of B cells, which includes memory B cells and plasma cells, is not readily detected in the thymus of infants or young mice but gradually accumulates during normal aging. Remarkably, in many autoimmune diseases the thymus suffers severe structural atrophy and infiltration of B cells in the perivascular spaces, which organize into follicles similar to those typically found in secondary lymphoid organs. This review provides an overview of the pathways involved in thymic B cell origin and presents an integrated view of both thymic medullary and perivascular B cells and their respective physiological and pathological roles in central tolerance and autoimmune diseases.

## 1 Introduction

Several decades ago, B cells were identified as a normal component of the human and mouse thymus (1, 2). Given their specific detection in the medulla it was acknowledged that they are a resident subset rather than B cells casually circulating through the thymus. Perhaps the most surprising aspect of thymic B cells is their unconventional origin through an intrathymic developmental pathway. Although B cells represent a small proportion of total thymocytes, they are a significant component in the medulla, occupying on average 30% of the area in that compartment (3), and are more abundant than thymic dendritic cells (3, 4). Their localization in the thymus and their intrinsic capacity to present antigens has driven much of the thymic B cell research to establish their involvement in central tolerance.

A generally overlooked aspect in studies of thymic B cells is the effect of aging on the thymic structure and cellular composition, which have mainly been conducted using infant thymic tissue and young mice. During the physiological process of thymic involution, there is an expansion of non-epithelial non-thymopoietic areas known as perivascular spaces (PVS). These PVS are not inert, they contain lymphocytes which are thought to be mature cells that recirculate back to the thymus (5). Surprisingly, many B cells accumulate in the PVS during normal aging, including IgG^+^ class-switched memory B cells and plasma cells with reactivity to common pathogens (3, 6). In several autoimmune diseases the recruitment of B cells to the PVS is exacerbated and often leads to the formation of germinal centers that sustain the differentiation of autoreactive plasma cells (1, 7–18). Thus, thymic B cells that accumulate in the PVS play a physiological role by maintaining protective humoral memory and a pathological role in the context of autoimmunity as a site where self-reactive antibodies are produced.

We review the current understanding of the unique properties of thymic B cells in mice and humans including their origin and development, phenotype, and various functions. We aim to propose a cohesive model in which the thymus is populated by two types of B cells: resident B cells located in the thymic medulla that appear in early life during fetal development that contribute to central tolerance as specialized APCs, and perivascular B cells that migrate from the periphery which play a more conventional role in the adaptive humoral response both in health and disease.

## 2 Origin of Thymic B Cells

**Figure 1:**
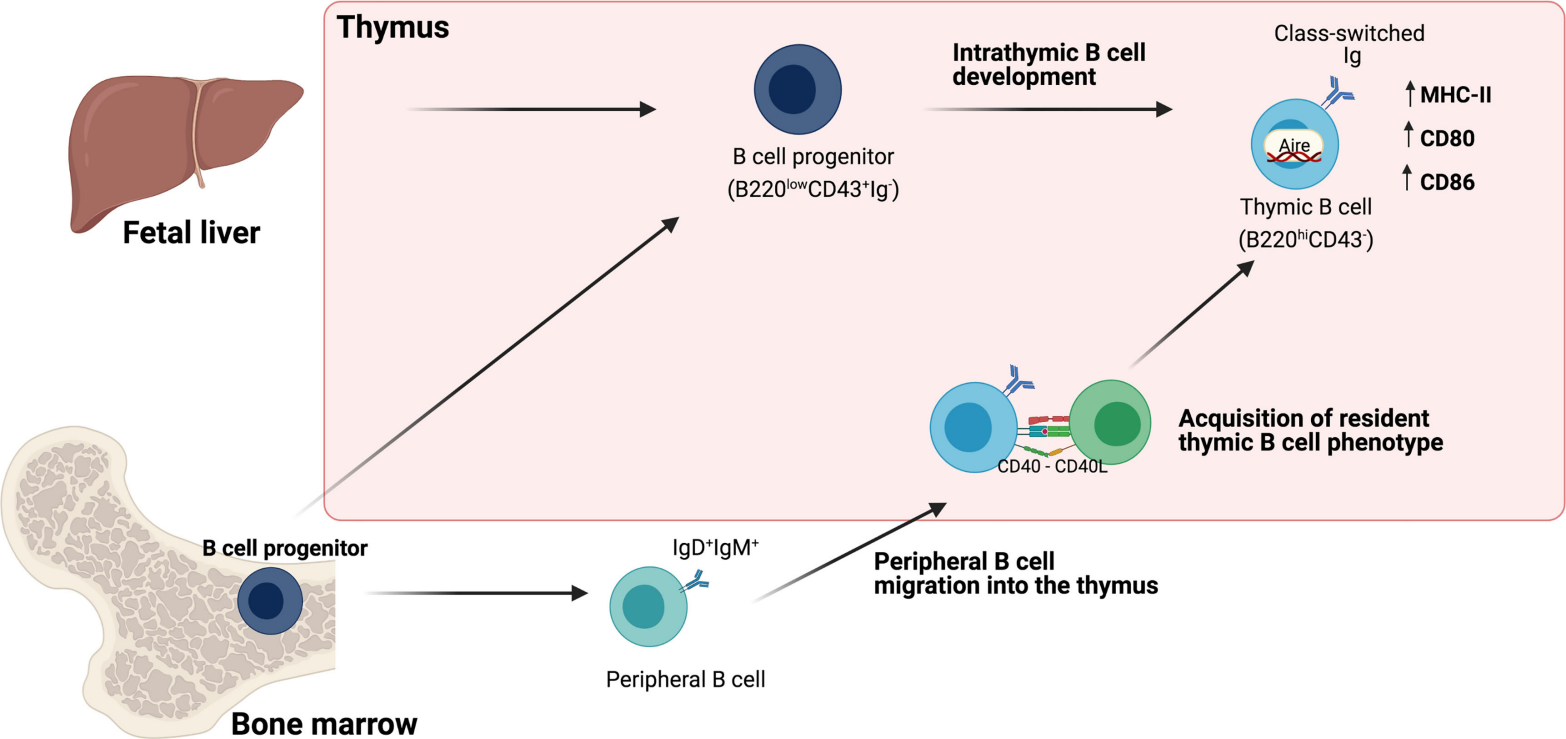
Origin of Thymic B cells. Schematic representation of different pathways involved in the origin of thymic B cells. One pathway proposes intrathymic differentiation from fetal liver and bone marrow B cell progenitors that give rise to resident thymic B cells. A second pathway is the migration of mature B cells, which acquire the phenotype of resident thymic B cells through the interaction with developing thymocytes (discussed in Section 2).

Though the presence of B cells in the thymus has been well established, their origin remains a matter of analysis. Some evidence suggests that, unlike the vast majority of B cells that differentiate in the bone marrow, thymic B cells differentiate within the thymus from early thymic precursors (ETPs) (8–10, 12, 13). However, it has also been shown that a small number of B cells can enter the thymus from the circulation and acquire a similar phenotype of thymic resident B cells (4, 19) (**Figure 1**).

### 2.1 Intrathymic B Cell Development

In the bone marrow, B cells arise from common lymphoid progenitors that undergo several stages of development. One of the earliest is the pro-B (progenitors of B cells) stage in which cells express some B-cell-specific markers such as B220 and CD19, but lack surface immunoglobulin (Ig) and other mature B cell markers. Pro-B cells initiate Ig heavy chain rearrangement and transition to the pre-B (precursors of B cells) stage characterized by the expression of a pre-BCR. Pre-B cells subsequently undergo Ig light chain rearrangement and become immature B cells that express a functional BCR. Immature B cells exit the bone marrow and reach the spleen, where they complete their development into mature B cells (20, 21). B cells colonize the thymus very early in life during fetal development. In mice, B220^+^ B cells are detected as early as gestational day 18, suggesting that the bone marrow is not essential for their development (22, 23). Moreover, in adult mice the thymus harbors populations of pro-B (B220^int^CD43^+^IgM^-^) and pre-B (B220^int^CD43^-^IgM^-^) cells similar to B cell progenitors that are normally found in the bone marrow (19, 24). Although B cell development classically occurs in the bone marrow and fetal liver, these findings suggest that thymic B cells could originate from precursors that differentiate locally. When thymic B cell progenitors (B220^int^CD43^+^) are isolated and reinjected into a thymus lobe of neonatal congenic mice, they can differentiate intrathymically into mature B220^+^IgM^+^ B cells (24). More recently, Perera et al, provided further evidence of intrathymic B cell maturation by using recombination-activating gene 2-GFP reporter mice to label B cells that have recently undergone BCR rearrangement. They identified B220^lo^CD43^hi^ cells positive for GFP which express lower levels of CD19 and MHC-II, indicating that they are equivalent to pre-B and pro-B rearranging their BCR. As expected, mature B220^hi^ cells were negative for GFP. A third population of B220^hi^ thymic B cells with intermediate GFP expression was also distinguishable, likely representing newly mature B cells that have recently completed BCR rearrangement. Consistently, BrdU labeling showed that immature B220^lo^GFP^+^ cells could divide rapidly, while mature B cells had a much slower turnover rate (25). Arguing against B cell development in the thymus, Yamano et al. compared the phenotype of putative thymic progenitors with those found in the bone marrow based on c-Kit expression. c-Kit, a receptor for stem cell factor is expressed at the pro-B stage and has an essential role in adult mice in the maintenance of B lymphopoiesis (26, 27). The authors observed that in the bone marrow CD19^+^IgD^-^IgM^-^ B cell progenitors can be subdivided into pre-B cells (CD2^+^cKit^-^) and pro-B cells (CD2^-^cKit^+^), that lack any Ig on their surface. However, among the CD19^+^IgD^-^IgM^-^ population in the thymus, none express a pro-B cell phenotype and most CD2^+^cKit^-^ express IgG on their surface, thus corresponding to mature class-switched B cells rather than progenitors (4). It is possible that B cell progenitors are scarce in the adult thymus and that their phenotype does not fully overlap with those found in the bone marrow.

In the human thymus, it is also possible to identify B cells from different developmental stages, including subsets with an immature phenotype equivalent to pre-B (CD10^+^IgM^-^CD34^-^) and pro-B (CD10^+^IgM^-^CD34^+^) cells usually found in the bone marrow (2); however, further studies are needed to address whether the human thymus can support B cell development.

It appears that some progenitors that colonize the thymus maintain B cell lineage potential; however, they are outcompeted by T cell progenitors in the thymic environment. Luc et al. reported that ETPs that seed the thymus for T cell development, also preserve their potential for B cell lineage differentiation in young and adult mice. The ETPs that maintain B cell lineage potential are contained in the Lin^-^CD4^-^CD8α^-^CD25^-^c-Kit^hi^Flt3^hi^ subset (28). Notably, the frequency of Flt3^hi^ ETPs is tenfold lower in the thymus of adult mice than in newborn mice (28). Moreover, when single Flt3^hi^ ETP cells are sorted and seeded onto a bone marrow stromal cell line, a lower frequency of Flt3^hi^ ETPs from adults is able to differentiate into B cells (28). Thus, the B cell potential of ETPs decreases with age. Other studies have also shown that thymic stromal cell lines support the development of mature IgM^+^ B cells from hematopoietic precursors *in vitro* (29, 30).When fetal thymic lobes are cultured in the presence of Flt3 and IL-7, progenitors expand and differentiate into mature and functional B cells, supporting that the fetal thymus provides an environment that can support the development of T and B cells (31). IL-7, which is essential for the development of T cells and γδ cells in the thymus, also influences the number of thymic B cells. Transgenic mice that overexpress IL-7 have significantly increased numbers of thymic B cells (32). A similar effect on the number of thymic B cells is observed in mice with conditional deletion of IL-7R in CD4^+^CD8^+^, CD4^+^ and CD8^+^ thymocytes (33). In the bone marrow IL-7 promotes B cell specification and commitment, survival of pro-B cells, and proliferation of pro-B and pre-B cells (34, 35). Therefore the balance between T and B lymphopoiesis is most likely controlled by the availability of IL-7 in the thymus. The frequency of thymic B cells is also increased in the thymus of mice that present a defect in T cell development such as TCR^-/-^ mice and Tgε26 transgenic mice, which have a blockade in the early development of thymocytes due to their expression of the human CD3E gene (19, 36, 37). This phenomenon is also observed in mice deficient in Notch-1 –an essential receptor that promotes T cells lineage commitment – where a decrease in T cells precursor is associated with an expanded number of thymic immature B cells (38, 39). While these studies suggest that the changes in thymic B cell frequency in these models are due to an increase in the lymphopoiesis of B cells as a consequence of a disruption of the T cell niche, a caveat that should be considered is that these models also present a significant decrease in thymic cellularity, which may result in a relative increase in the frequency of thymic B cells due to overall loss of thymocytes.

### 2.2 Migration of Circulating B Cells to the Thymus

Another alternative that would explain the origin of B cells is the migration of mature peripheral B cells to the thymus. When splenic B cells are transferred intravenously to a congenic mouse to evaluate what percentage of B cells from the donor is capable of migrating into to thymus and other organs, at 18 days post transfer it was observed that only 0.6% of the donor-derived cells migrated to the thymus, while about 5% of the total lymph node and spleen cells came from the donor (19). Similarly, Yamano et al. reported that the frequency of transferred splenic B cells migrating to the thymus was 5-10-fold lower than the spleen (4). In parabiosis assays, which allow a more physiological study of the migration of circulating B cells to the thymus, it was observed that about 50% of mature B cells in the spleen were donor-derived cells, rapidly equilibrating, while the thymus showed a lower exchange where only 20% of mature B220^+^ cells come from the donor (25, 40). These studies conclude that there is a contribution to the thymic B cell pool through the migration of peripheral B cells, although it appears to be minimal in mice. Inefficient recirculation of mature lymphocytes to the thymus is thought to be partly due to limited available niches to accommodate immigrating cells. The recirculation of B cells to the thymus has been studied in young mice in which thymic involution is not significantly noticeable. It is possible that if similar studies were performed in aged mice the proportion of B cells derived from circulation would be greater. In agreement with this, Cepeda et al. find a significant increase in the frequency of thymic B cells when mice are at least 12-24 months old and show and evident thymic decline (41). When thymic cellularity is reduced by inducing the death of thymocytes through irradiation or acute involution triggered by systemic LPS treatment, enhanced migration of peripheral B and T cells occurs (4, 42). In LPS-treated mice, the MCP-1/CCR2 axis is one of the mechanisms that directs the migration of peripheral B cells (42). This suggests that in the under inflammatory or infectious conditions, thymic involution combined with the induction of chemokines can drive the accumulation of peripheral B cells in the thymus. Further evidence of this is the abnormally expanded frequency of thymic B cells in autoimmunity (discussed in Section 5). Altogether, we still lack a clear understanding of how the thymic B cell population is maintained over time and the relative contribution of intrathymic differentiation from precursors, self-renewal and import of peripheral B cells to the thymic B cell pool.

## 3 Phenotype of Thymic B Cells

### 3.1 Mouse Thymic B Cells

Thymic B cells have an unusual phenotype compared to circulating and secondary lymphoid organ B cells. Initial analysis of thymic B cells in mice revealed a high expression of CD5 and CD43, which are hallmark surface markers of the B1-a lineage located predominantly in the peritoneum, suggesting that thymic B cells may arise from fetal liver progenitors (43, 44). However, a more careful comparison of bona fide peritoneal B1 cells and thymic B cells shows a non-overlapping pattern in the expression of CD5 and CD43, which is lower and less well-defined in thymic B cells. Also, thymic B cells unlike peritoneal B1 cells lack CD11b (25). Moreover, when RAG^-/-^ mice are lethally irradiated and reconstituted with cells from either fetal liver or bone marrow, both can partially restore thymic B cells, albeit not to their normal numbers. In contrast, peritoneal B cells are fully restored by the fetal liver but not bone marrow cells (25). Thus, it appears that thymic B cells and B1 B cells are from different lineages and that bone marrow and fetal liver cells contribute to the development of thymic B cells. Analysis of CD21/CD23, which distinguishes classic splenic subsets, shows that thymic B cells are composed of CD21^+^CD23^+^cells that resemble follicular B cells and CD21^-^CD23^-^ B cells but do not contain CD21^++^CD23^-^ marginal zone B cells (25).

Thymic B cells also differ from resting peripheral B cells in that under steady-state conditions, they display an activated phenotype, expressing high levels of MHC-II as well as significantly higher levels of costimulatory molecules CD80, CD86 compared to B cells from the spleen (4, 25, 43, 45). Most thymic B cells express CD69 and low levels of CD62L, which have been associated with an early activation state (43, 45). However, recent studies show that resident memory B cells located in lymphoid and non-lymphoid tissues are also characterized by a CD69^+^CD62L^lo^ phenotype (46–48). Given that thymic B cells are not readily exchanged with circulatory B cells, it is likely these markers indicate that thymic B cells are resident, as opposed to recently activated, cells.

### 3.2 Intrathymic B Cell Licensing and the Central Role of CD40/CD40L Interaction

An unexpected property of thymic B cells is that in mice without prior infection or immunization about one-third are antigen-experienced IgD^-^IgM^-^ class-switched B cells and express other subtypes of IgG and IgA on their surface, IgG2b being the most frequent, while most peripheral B cells are naïve IgD^+^IgM^+^ (4, 40).

A pivotal study by Yamano et al. demonstrated that the phenotype of B cells is modified within the thymus through a process recently termed as thymic licensing. When naïve B cells are transferred intravenously, they retain their original phenotype in the spleen; however, those that immigrate into the thymus acquire the features of resident thymic B cells, including increased levels of MHC-II and co-stimulatory molecules CD80 and CD86, thus enabling them to act as more efficient APCs (a function that will be discussed later). Furthermore, they also identified a fraction of thymic B cells that undergo immunoglobulin class-switch and acquire the expression of the autoimmune regulator (Aire) gene (4). Aire is classically induced in medullary thymic epithelial cells (mTEC), allowing these cells to express autoantigens in the thymus that are only found in peripheral tissues promoting self-tolerance to a broad range of autoantigens (49). However, Aire protein levels are significantly lower in B cells than mTECs and Aire regulates fewer genes in thymic B cells (4). Most importantly, the same programming of the B cell phenotype occurs when naïve B cells are injected directly into the thymus, reinforcing that licensing is elicited by local cues (4).

Thymic B cells differ in their responsiveness to conventional B cell stimuli compared to splenic B cells. They do not proliferate or differentiate into antibody-secreting cells to the same extent as splenic B cells in response to LPS or anti-IgM+IL-4. However, they are significantly more responsive when they interact with activated T cell blasts (50), or are activated by a combination of anti-CD40 and IL-10 (24, 51). This superior response to CD40 stimulation is also demonstrated in the strong requirement of CD40/CD40L signaling to maintain a normal frequency of thymic B cells provided through interaction with developing T cells. Mice lacking either CD40 or CD40L have significantly reduced numbers of thymic B cells while maintaining a standard B cell frequency in peripheral lymphoid organs (52, 53).

Notably, cognate interaction with developing thymocytes provides necessary signaling for intrathymic B cell licensing, i.e., higher levels of MHC-II, induction of Aire, and isotype class-switching (4, 40). Thymic B cells fail to acquire these features under conditions where antigen presentation to CD4 SP thymocytes is blunted, such as a lack of MHC-II on B cells, CD40 deficiency, as well as in TCRα^-/-^ mice. Licensing is also altered in OT–II mice indicating that a normal T cell repertoire and cognate B-T interaction is required (4, 40).

### 3.3 Human Thymic B Cells

Few studies have analyzed the phenotype of human thymic B cells, mainly through immunohistological methods rather than more comprehensive flow cytometry. These reports show that B cells in the human thymus, similar to mice, are found in a small proportion presenting a unique phenotypic characteristic compared to peripheral B cells. About 50% of thymic B cells express CD5 on their surface, while peripheral blood B cells are mostly CD5^-^ (54). In the human thymus, B cells uniformly express maturation markers such as CD19, CD22, CD20, CD40, and CD2 (22, 55–57). About 10% express Ki-67, indicating that some thymic B cells are actively proliferating (22, 55, 57). Work from our group found that human thymic B cells also display elevated levels of MHC-II, CD80, CD83, and CD86. Notably, in young infants, thymic B cells consistently express higher amounts of MHC-II molecules than thymic CD11c^+^ dendritic cells, suggesting that they are poised to present antigens efficiently, similar to what has been as reported in mice (3).

In a longitudinal analysis of the distribution and phenotype of human thymic B cells spanning seven decades of life, our group also demonstrated the existence of two distinct subsets of B cells in the human thymus located in the medullary and the perivascular compartments respectively. Medullary B cells co-localize with mTECs and immature thymocytes. Medullary B cells represent the vast majority of thymic B cells in neonates and infants, yet their relative proportion decreases as the thymic epithelial areas decline in adulthood (3). The second subset of B cells begins to appear in the thymic PVS after the first year of life, which expands with age, allowing an increasing number of B cells to accumulate in this area (discussed in Section 4). PVS B cells are primarily composed of class-switched IgG^+^CD27^+^ memory cells, the main subset of thymic B cells in adults. Terminally differentiated plasma cells that secrete primarily IgG also begin to populate the PVS during childhood and are maintained in adults (3).

It is noteworthy that in infants where most thymic B cells are located in the medulla, even in the first weeks of life, between 15-30% have already undergone Ig class-switch to IgG or IgA (3). Such observation warrants further research to determine whether human B cells also go through an equivalent thymic licensing process as described in mice.

## 4 Thymic B Cell Functions

### 4.1 The Role of Thymic B Cells in Central Tolerance

**Figure 2:**
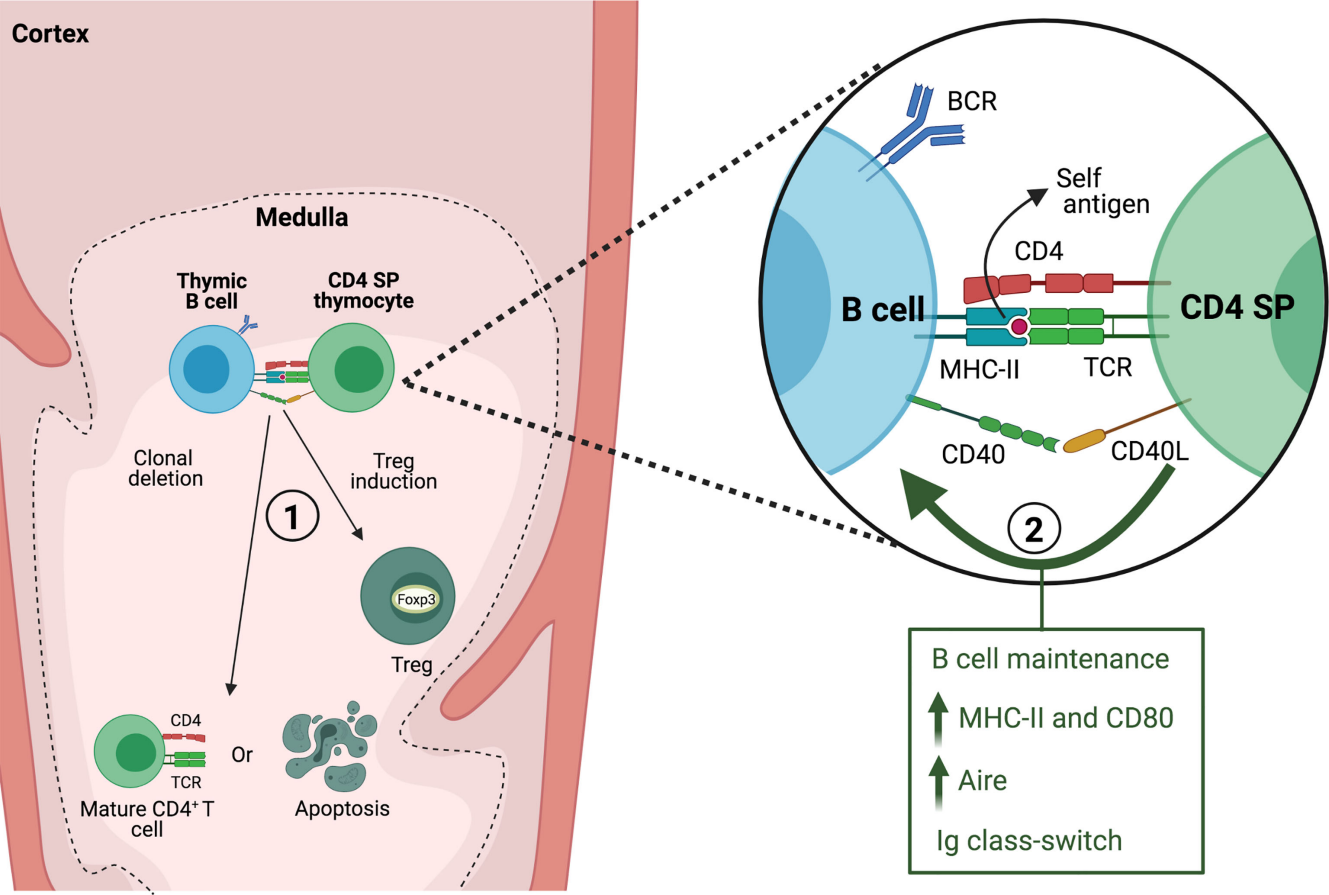
Interaction between thymic B cells and CD4 SP thymocytes. In the thymic medulla, B cells present self-antigen to CD4 SP thymocytes through MHC-II and engagement of CD40/CD40L. The interaction between both cells leads to 1) clonal deletion or generation of thymic regulatory T cells (Treg) and 2) is required for maintaining normal thymic B cell numbers and acquisition of a thymic resident B cell phenotype.

Several experimental models have demonstrated that thymic B cells play a non-redundant role in the negative selection of self-reactive clones (**Figure 2**). This function was first investigated using mouse strains bearing different Minor lymphocyte stimulating antigens (Mls). Neonatal intrathymic injection of thymic B cells that express Mls-1 antigen results in a reduced frequency of Mls-1 reactive vβ6^+^ thymocytes, however Mls-1+ splenic B cells are unable to effectively induce clonal deletion either *in vivo* or in fetal thymus organ culture (58, 59). This suggests that thymic B cells induce clonal deletion of self-reactive thymocytes, although it should be considered that this effect may be partly attributed to the presentation of Mls-1 antigen by endogenous dendritic cells. In turn, T cells from mice that have been tolerized with Mls-1 thymic B cells have a restrained ability to mount a graft-versus-host response when transferred into Mls-1-bearing mice (58). Likewise, thymic B cells expressing viral superantigen Mtv-7 effectively mediate clonal deletion of MtV-7-reactive transgenic thymocytes *in vitro*, whereas splenic B cells showed weaker capacity unless previously activated through CD40 engagement (43). These early studies suggested that thymic B cells might be conditioned by their environment to enhance their ability to interact with cognate T cells and participate in negative selection.

More recent studies have established the contribution of endogenous thymic B cells to central tolerance. In transgenic mice in which I-E molecules are expressed exclusively by B cells, there is significant deletion of I-E-reactive vβ5 and vβ11 T cell clonotypes (60). B^MOG^ mice which have forced presentation of a Myelin oligodendrocyte glycoprotein (MOG) peptide on MHC-II by B cells are tolerized against induction of EAE (61), which suggested the ability of B cells to purge MOG-reactive T cells. This was corroborated by generating 2D2/B^MOG^ mice, bearing a MOG-specific TCR, in which a drastic reduction of thymic and peripheral transgenic CD4^+^ T cells was observed along with a high frequency of Tregs among the surviving self-reactive T cells (62). Perera et al. used a novel approach to understand how reactivity towards self-antigens in the thymic B cell repertoire may favor their ability in negative selection. Mice in which B cells express a transgenic BCR with specificity for a self-antigen display enhanced negative selection of cognate CD4 SP thymocytes. They also found that when mice express a self-reactive transgenic TCR, a lack of B cells results in an incomplete negative selection, indicating that the endogenous B cell repertoire contributes to this process (25). There is evidence that autoreactive B cells are enriched among thymic B cells in mice and humans (40, 63). By cloning Ig genes from single cells and testing their reactivity against a panel of self-antigens it was determined that significantly more antibodies derived from human thymic B cells show reactivity to nuclear antigens from a Hep-2 cell lysate compared to B cells from bone marrow. A high frequency of thymic antibodies also binds to dsDNA, while only few recognize insulin peptide or malondialdehyde-modified BSA (an oxidized lipid antigen found in apoptotic cells) (63). Though it is not clear what types of self-antigens could recognized by thymic B cells, it has been proposed that a broad recognition of self-antigens would enhance their capacity to purge cognate thymocytes. In mice, a bias towards autoreactivity (i.e., binding to nuclear antigens in Hep-2 cells) occurs among Ig class-switched thymic B cells, consistent with an enhanced capacity to present self- antigens, interact strongly with thymocytes and undergo thymic licensing (40). Additionally, Yamano et al. have demonstrated that self-antigens induced by Aire specifically in B cells, therefore dependent on thymic licensing, can also drive clonal deletion of cognate CD4 SP thymocytes (4).

A distinct property of thymic B cells is that they are the first antibody-secreting cells (ASCs) that spontaneously secrete IgG, IgA and IgE which appear early in the postnatal thymus, before to the appearance of ASCs in the spleen (64, 65). In a pig model, thymic B cells are also the first to undergo Ig class-switch and secrete IgG and IgA spontaneously during the gestational period and in newborn germ-free piglets, whereas splenic B cells only secrete IgM (66–68). Moreover, colonization of germ-free piglets with *E. coli* leads to IgG and IgA diversification in the mesenteric lymph node and blood and selective expansion of clones. In contrast, the thymic B cell repertoire remains polyclonal and unaffected by colonization (68). Therefore, thymic Ig class-switch and secretion do not appear to be driven by external antigenic stimuli o microbiota colonization. One explanation for why Ig class-switch and secretion may be functionally relevant in the thymus is to establish T tolerance to immunoglobulins. In the case of IgE, Haba et al. show that mice become tolerant to IgE, i.e., fail to generate anti-IgE antibodies when immunized, at postnatal day 10, which coincides with the appearance of IgE ASCs in the thymus (65). Also, during the generation of a humoral response, B cells internalize, process and present antigen-derived peptides. In this process, peptides from the BCR are also processed and presented, therefore, establishing T cells tolerance to such antigens is critical to avoid inadequate and potentially harmful activation of immunoglobulin-specific T cells. Thymic B cells have been shown to negatively select TCR-transgenic T cells that recognize Ig determinants, therefore ensuring tolerance for subsequent cognate B-T interaction (69, 70).

Thymic B cells have also been shown to contribute to the differentiation and expansion of thymic Tregs. Transgenic BAFF mice, which have a 7-fold increase in the frequency of thymic B cells, have twice as many Tregs, whereas, in B cell-deficient uMT mice, Tregs are reduced by nearly half (45, 71). Histological staining shows that B cells are co-localized with Tregs in the thymic medulla, and they can induce the differentiation of CD4 SP thymocytes intro Tregs *in vitro* (45). The expansion of thymic Tregs requires cognate B cell help through MHC-II molecule as well as a polyclonal BCR repertoire, suggesting that this interaction may be initiated after self-antigen uptake, processing, and presentation by thymic B cells (45, 71). In the human thymus, IL-15 promotes the differentiation of Treg precursors into mature Foxp3^+^ Tregs and the proliferation and survival of Tregs. Thymic B cells express high levels of IL-15 mRNA compared to other thymic subsets and co-localize in IL-15-rich areas in the thymic medulla, therefore B cells may promote Treg differentiation through the production of this cytokine (72).

Thymic B cells also appear to regulate normal mTEC development and expression of tissue-restricted antigens (TRAs) partly through the production of lymphotoxin α (LTα) and β (LTβ). Akirav et al. have shown that LTα and LTβ mRNA is expressed by thymic B cells in addition to CD4 and CD8 SP thymocytes. Moreover, they observe that the number of mature mTEC is reduced nearly by half in mice with diminished numbers or lacking thymic B cells. μMT mice lacking B cells also exhibit a lower expression of Aire and TRAs such as insulin and MOG in the thymus. Notably, in mice with conditional deletion of LTβ expression specifically in B cells, there is also a reduced expression of TRAs, suggesting that LTβ deficiency in B cells impairs mTEC development (52, 73).

### 4.2 Perivascular B Cells: A Niche of Protective Humoral Memory

**Figure 3:**
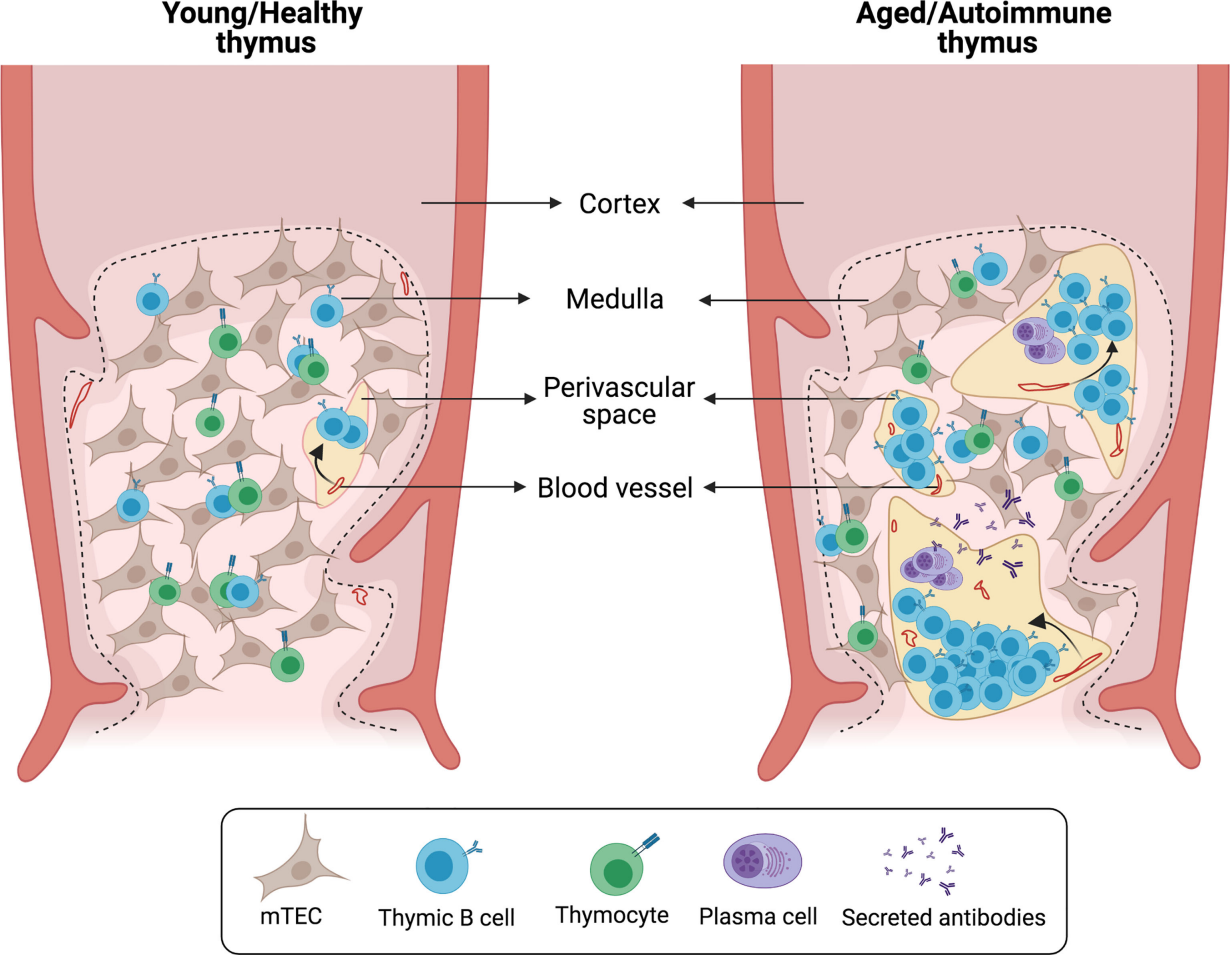
Histological changes in aged/autoimmune thymus. Schematic representation of characteristic alterations in the aged/autoimmune thymus. The medullary epithelial network is disrupted by the enlargement of perivascular spaces (PVS). B cells and plasma cells infiltrate the thymus and localize within the PVS forming germinal center-like structures.

As mentioned previously, numerous B cells are also found in the human thymus PVS (3, 5, 6). However, to date few studies have investigated the nature of these B cells. The area of the thymus occupied by PVS progressively increases with age, starting to expand from early childhood (3, 5). These structural changes are accompanied by an increased frequency of thymic B cells in older children and adults (3, 6). Immunohistochemical staining of several B cell markers combined with cytokeratin staining shows that most PVS B cells express CD21, CD72 and CD37, while these markers are absent in medullary B cells (6). These phenotypical differences would support that medullary and PVS B cells are separate subsets with little or no exchange. After the first year of life, the accumulation of B cells in PVS correlates with an age-associated increase in the frequency of CD27^+^IgG^+^ memory B cells, suggesting that most PVS B cells consist of memory B cells (3). Furthermore, a significant proportion of B cells that accumulate in the thymus during aging express the chemokine receptor CXCR3, suggesting that PVS B cells migrate to the thymus after being activated in the periphery (3). CD138^+^ plasma cells are also found in the thymic PVS and secrete IgG without the need for additional stimulation. Thymic plasma cells secrete primarily IgG1 and IgG3, the two main complement-fixing effector IgG subclasses. Antigen-based ELISPOT assays show that human thymic plasma cells contain a high frequency of cells reactive to common viral antigens encountered through infection and vaccination, including influenza, rubella and measles (3). Notably, the PVS also harbors eosinophils that are located in close proximity to plasma cells (3, 5). Eosinophils are essential for the maintenance of long-lived plasma cells in the bone marrow and gut-associated tissue (74, 75), therefore the PVS may acts as a functional niche for viral-specific plasma cells (**Figure 3**).

### 4.3 Aging and Thymic B Cell Function

During aging, immune responses against new antigens are diminished while the risk for developing autoimmune disease generally increases (76). Thymic involution, a hallmark of aging, is characterized by a steady loss of cellularity and a decline in the export of mature T cells. Age-associated functional decline in the thymus is also believed to be related to the increased susceptibility to autoimmune disorders (77). Since B cells play a critical role in the induction of central tolerance, a relevant question is whether a decline in the function of thymic B cells could contribute to defects in T cell selection. We have found that aging significantly impacts the phenotype of human thymic B cells, particularly in regard to their antigen-presenting function. We determined a nearly 10-fold reduction in the expression of MHC-II between infants and adults, and a significant reduction in the levels of co-stimulatory molecules CD80, CD83, and CD86 (3). In addition, Cepeda et al. recently reported that while the relative proportion of B cells increases in aged mice, the expression of Aire declines in thymic B cells, including in IgD^-^IgM^-^ class switched cells that have presumably undergone licensing (41). Transcriptional analysis of human thymic B cells from young and aged individuals also shows diminished Aire expression. The observed loss of Aire expression is accompanied with changes in the expression of Aire-dependent genes both in mice and humans (41). These findings suggest that the ability of thymic B cells to enforce central tolerance is dampened by aging.

## 5 Autoimmunity-Driven Accumulation of B Cells in the Thymus

In several autoimmune diseases and relevant animal models, there is a drastic deterioration of the thymic epithelial network associated with elevated numbers of B cells that accumulate in the PVS and organize into follicles and active germinal centers (**Figure 3**). Depending on the disease and its severity, thymic alterations can range from a modest increase in the frequency of thymic B cells to a complete remodeling of the thymus into what resembles a secondary lymphoid organ (**Table 1**). In this section we will review the diseases in which concomitant thymic pathology has been described.

**Table 1:** Various autoimmune disease and relevant animal models that are associated with thymic pathology.

	Thymic pathology			Thymic pathology	
Disease	Thymic stromal changes	Cellular changes	Animal model	Thymic stromal changes	Cellular changes
Myasthenia gravis	Epithelial areas are replaced by perivascular spaces (8) Higher expression of CXCL13 and BAFF in TECs (78, 79) Increase of lymphatic vessels and high endothelial venules (HEV) (80, 81)	Formation of germinal centers containing activated B cells (1, 7, 82) Plasma cells that produce autoantibodies against AchR (83–85)	K5-CXCL13 mice immunized with AchR (86)	Not specified	Recruitment of B cells in the thymus (86)
SLE	Severe atrophy, loss of the cortex and disorganization of epithelial cells in the medulla (10)	Germinal centers in a reduced subset of patients (9, 10) Increased number of plasma cells (9, 10)	BWF1, MRL/MP-lpr/lpr, BXSB/MpJ Yaa and C3H HeJ-gld/gld mice (87–91)	Loss of normal stromal structure with a disorganized network of epithelial cells (87–91) Enlarged PVS (11, 92, 93) High expression of CXCL13 in myeloid DCs (93, 94) Expression of PNAd in blood vessels in PVS (93)	B cells organized into germinal center-like structures (11) Increased frequency of TFH cells (11) Plasma cells producing anti-dsDNA IgG (11)
T1D	Not studied	Not studied	NOD mice (12, 95–97)	Enlarged PVS (95–97)	Increased number of B cells, plasma cells and TFH cells (12) Formation of ectopic germinal centers (12)
Sjogren’s syndrome	Severe atrophy with formation of thymic epithelial cysts (13–15, 98)	Germinal centers associated to thymic epithelial cysts (13–15)	IQI/*Jic*, and Aly/*aly* mice (99)	Disorganized stromal network (99)	Increased number of B cells (99)
Ulcerative colitis	Not specified	Formation of lymphoid follicles (16)	DSS induced colitis, Gαi2^-/-^ mice (100–102)	Acute thymic involution (100–102) Loss of CD4^+^CD8^+^ thymocytes (100–102)	Not studied
Rheumatoid arthritis	Thymic hyperplasia; low-grade B cell lymphoma (17, 18, 103)	High density of B cells, germinal centers, plasma cells (17, 18, 103, 104)	–	–	–

### 5.1 Myasthenia Gravis

The most studied disease that is characterized by its associated thymic pathology is myasthenia gravis (MG). Approximately 60-80% of MG patients develop a condition defined as thymic follicular hyperplasia in which the normal thymic lymphoepithelial architecture is disrupted and replaced by B-cell follicles containing active germinal centers that give rise to plasma cells (1, 7, 82). The incidence of thymic pathology reported in MG patients varies in the literature due to an evident gender and age bias (8). Thymic pathology occurs mainly in females and peaks between the ages of 20-40 years. The number of thymic germinal centers wanes in patients over the age of 50 years when the lymphoepithelial areas have been broadly replaced by adipose and connective tissue, which likely fails to provide a favorable environment for GC maintenance (8). The thymus of MG patients contains activated B cells undergoing clonal proliferation and hypermutation and plasma cells that produce autoantibodies against the acetylcholine receptor (AChR) (83–85). Consequently, the incidence of thymic B cell germinal centers correlates with anti AChR titers (8). Converging evidence that the thymus is actively contributing to the pathogenesis in MG has been validated in retrospective analysis and, more recently, a randomized clinical trial of thymectomy, which shows significant improvements in the clinical outcome after surgery compared to steroid therapy alone (82, 105).

Several mechanisms at play in the development of thymic pathology in MG have been identified. The abnormal recruitment of B cells results from an elevated production of the B-cell attracting chemokine CXCL13 and B-cell activating factor (BAFF) by thymic epithelial cells (78, 79). IFN-β, a signature cytokine in many autoimmune diseases, can induce the expression of CXCL13 and BAFF in TECs (78). A possible scenario is that chronically elevated levels of inflammatory cytokines condition the thymic environment to promote the recruitment of B cells. Transgenic mice in which mTECs overexpress CXCL13 have normal numbers of thymic B cells, however, inflammation induced by the administration of TLR3 agonist Poly(I:C) promotes infiltration of B cells in the thymus and increases the susceptibility of severe MG when they are immunized against AChR (86). Therefore, thymic pathology requires a combination of increased B cell chemotactic signals in the thymic environment as well as B cell activation in response to systemic inflammation.

Another key feature of thymic follicular hyperplasia in MG patients is the increased development of lymphatic vessels and high endothelial venules (HEVs) (80, 81). Blood vessels associated with follicular cell aggregates originate in the PVS and become equipped with the tall endothelium of the post-capillary-venule type. In physiological conditions HEVs are found in secondary lymphoid organs, yet they can also appear in chronically inflamed tissues where they are associated to the infiltration of peripheral cell aggregates (106). Therefore, the presence of HEVs in the MG thymus suggests active immigration of lymphocytes from the bloodstream.

Conventional corticosteroid treatment normalizes several features of thymic pathology in MG patients, reducing the number of germinal centers, the expression of chemokines that orchestrate GC formation, including CXCL13, CCL21 and CCL19 (79, 107), and also the number of HEVs (81). These findings are not trivial as they strongly argue that thymic disturbances are a consequence rather than a cause of autoimmune disease.

### 5.2 Systemic Lupus Erythematosus

Thymic abnormalities have been also been reported in patients with systemic lupus erythematosus (SLE). The most representative manifestation is a high frequency (6-fold increase) of plasma cells compared to the thymus of both healthy subjects and MG patients. While some SLE patients also develop germinal centers in the thymus, it is not a defining feature as in MG (9, 10). The thymus also presents signs of extreme atrophy in SLE patients with complete loss of the cortex and disorganization and aggregation of epithelial cells in the medulla (10).

Although there are limited human studies to understand the underlying causes and implications of thymic pathology associated with SLE, it has been further examined in mouse strains that spontaneously develop a systemic autoimmune disorder that resembles human SLE. Early histological studies in the thymus of NZB, BWF1, MRL/MP-lpr/lpr, BXSB/MpJ Yaa and C3H HeJ-gld/gld mice revealed that these models recapitulate a progressive loss of the normal stromal architecture. Typical stromal alterations include a disorganized network of epithelial cells and enlarged perivascular spaces that are densely packed with lymphocytic follicles and plasma cells (87–91). Later works have examined more closely the abnormal recruitment of B cells to the thymus. In aged BWF1 mice, the group of Ishikawa reported a 2000-fold increase in the expression of CXCL13 in the thymus. The primary source of CXCL13 is myeloid CD11c^+^ dendritic cells which are also more abundant in the thymus of aged mice. They found that B cells that accumulate in the thymus are composed of B1 (B220^int^CD5^+^) and B2 (B220^+^CD5^-^) B cell subsets and suggested that B1 cells may be preferentially recruited due to a higher expression of CXCR5 (94). The same group also demonstrated that in healthy mice B cells do not actively immigrate into the thymus, however; in diseased BWF1 adoptively transferred B cells are readily detectable in the thymus and mainly localize in the perivascular spaces (92, 93). Trafficking of peripheral lymphocytes to the perivascular spaces is supported by the expression of peripheral node addressin in localized blood vessels (93).

The degree of thymic structural alterations in BWF1 mice correlates with the levels of anti-dsDNA autoantibodies (91), however, a pivotal question to be addressed is the contribution of the thymus to the progression of the disease. We have recently shown that in diseased BWF1 mice, the thymus contains large numbers of B cells organized into germinal center-like structures as well as a distinct subset of follicular helper T cells, reminiscent of active germinal centers found in the thymus of MG patients. Moreover, we detected antibody-secreting cells in the thymus of diseased-BWF1 mice that produce anti-dsDNA IgG autoantibodies (11). Therefore, the thymus may constitute an important niche that supports the maintenance of the pathogenic humoral response in SLE.

### 5.3 Type 1 Diabetes

Type 1 diabetes (T1D) is a condition that results from the attack of pancreatic beta cells by innate and adaptive immune cells. How the disease progresses, and its underlying immunological mechanisms have been possible to study using the non-obese diabetic (NOD) mouse strain. Several studies aiming to understand whether thymus dysfunction contributes to immune dysregulation observed an accelerated deterioration of epithelial organization and formation of giant perivascular spaces (95–97). Furthermore, a recent study found that in NOD mice B cells begin to accumulate in the thymus at the prediabetic stage. B cells accumulate in follicle-like structures in non-epithelial areas which likely correspond to enlarged PVS described in earlier studies (12). In addition, they found that the NOD thymus contained significantly more follicular helper T cells and a slight increase in the number of plasma cells, altogether suggestive of the formation of ectopic germinal centers in the thymus (12). It remains to be studied if B cell accumulation in NOD mice is driven by similar changes in the thymic environment as those observed in MG and SLE, such as the production of CXCL13. The reactivity of B cells and plasma cells that infiltrate the thymus in NOD mice also need to be addressed, since it is not clear whether thymic abnormalities contribute to T1D progression or it is rather an epiphenomenon that occurs as a byproduct of peripheral B cell activation. A possibility is that in T1D, the thymus also harbors pathogenic B cells or plasma cells. In this sense, autoreactive islet antibodies are present in individuals that develop T1D. However, the pathogenicity of these antibodies is unclear, since they are dispensable for disease development in NOD mice (108). Nonetheless, B cells do play a critical role in T1D pathogenesis as antigen-presenting cells (108). In this regard, the capacity of B cells to interact with and potentially stimulate autoreactive T cells in the PVS is yet to be explored.

### 5.4 Other Diseases

Several case reports have followed patients with Sjogren’s syndrome requiring extended thymectomy due to the presence of multilocular thymic cysts in which thymic medullary epithelia undergo a cystic transformation as a result of inflammation (98). Thymic cysts are associated with lymphoid tissue containing active germinal centers (13–15). Furthermore, an increased number of thymic B cells and disorganized thymus structure have been observed in IQI/*Jic* and Aly/*aly* mouse strains that model Sjogren’s syndrome (99).

Thymic abnormalities have also been noted in patients with rheumatoid arthritis (RA). A clinical report of an RA patient not receiving corticosteroid therapy found that the thymic cortex was completely absent and identified active germinal centers (104). In other case reports, patients with RA and juvenile idiopathic arthritis develop thymic hyperplasia diagnosed as low-grade B cell lymphoma. In these patients, thymus biopsy reveals increased density of CD20^+^ B cells, lymphoid follicles with germinal centers and plasma cells scattered in the tissue (17, 18, 103).

Thymic pathology has also been observed in patients with ulcerative colitis. One study reported the presence of lymphoid follicles in 40% of UC patients (16). In a subset of patients with poor response to conventional treatment, close to 90% went into remission after thymectomy. indicating a contribution of the thymus to disease severity (109). Ulcerative colitis can be induced in mice by the administration of dextran sulphate sodium or occurs spontaneously in Gαi2^-/-^ mice. In both models the thymus undergoes atrophy associated with a dramatic reduction of double positive thymocytes (100–102), however, other alteration in the thymic organization and infiltration of B cells have not been addressed.

## 6 Challenges and Future Perspectives

Thymic B cells have been studied for several decades yet their unconventional nature still leaves many open questions. The process of thymic B cell licensing is particularly intriguing because it is not fully understood if thymic B cell isotype switching is driven by antigen encounter, or occurs in a random manner as a result of strong T cell help signaling. On average one third of thymic B cells have switched to IgG and IgA, yet it is not clear what determines that a thymic B cells will either retain IgD^+^IgM^+^ expression or undergo class-switch. Moreover, whether switched Ig regulate other properties of thymic B cells such as their survival or the ability to present antigens found in low concentration needs to be elucidated.

Although there is substantial evidence that B cells reside in two different compartments of the thymus, the medulla and PVS, few studies have addressed their coexistence or whether there is any exchange between them. The thymic B cell pool is the result of local differentiation and import from circulation, therefore a reasonable hypothesis is that medullary B cell arise primarily from B cell progenitors while PVS B cells are exclusively derived from circulating mature B cells. Analysis of the Ig repertoire between both subsets could provide insight into their different clonal origin and identify any overlapping clonotypes if exchange does in fact occur.

The balance between medullary B and PVS B cells evolves during the normal aging process and in autoimmune diseases. Starting from early childhood there is a decline in the area occupied by the medulla which is progressively replaced by the PVS, which occurs in an accelerated manner in individuals with autoimmune conditions. This has important implications for studies that aim to examine how aging and autoimmunity affects the phenotype and functionality of thymic B cells, as it requires that a distinction be made between medullary and PVS B cells. A recent study concluded that aging was associated with a decline in Aire expression and other genes such as CD80 (41). In our own studies we have found that thymic B cells in adults have lower levels of MHC-II (3). It is conceivable that this is actually a reflection of an increased ratio of PVS B cells, exhibiting have a different phenotype than medullary B cells. Future studies should consider the relevance of distinguishing both types of thymic B cells, for instance, through intravenous labeling in mice which stains cells in the PVS while sparing other thymic compartments (110).

Accumulation of B cells with reactivity to commonly encountered viruses during healthy aging and self-reactive B cells in MG and SLE strongly suggests that the PVS is a target niche for activated B cells, memory B cells and plasma cells generated during a physiological and pathogenic immune response. The development of chronic inflammation in autoimmune disease may amplify the mechanisms that normally regulate B cell recruitment to the thymus, thus it may be relevant to define common pathways, i.e. chemokines, cytokines, that are involved in both situations.

## 7 Conclusion

As our understanding of thymic B cells advances, it is evident that they cannot be regarded as a single homogeneous population. Different subsets are positioned in separate regions of the thymus, and most likely have a distinct origin, phenotype, and function. In this Review we propose that thymic B cells should be classified as medullary and PVS B cells. Medullary B cells populate the thymus early in ontogeny, originate mainly from intrathymic B cell differentiation of progenitors, and exert the role of specialized antigen-presenting cells in central tolerance. Remarkably, the thymic environment, in particular cognate interactions with immature thymocytes, appears to enhances the capacity of thymic B cells to mediate negative selection. On the other hand, PVS B cells populate the thymus later in life and accumulate gradually throughout aging. They are most likely peripheral B cells that migrate to the thymus, and include memory B cells and plasma cells. The presence of antiviral thymic plasma cells suggests that the PVS compartment may resemble the bone marrow as a functional niche of humoral memory. Future studies are urgently needed to fully understand the role of PVS B cells. Finally, the mechanisms by which the thymic PVS fosters antigen-experienced B cells and plasma cells appear to be detrimentally amplified in some autoimmune diseases. Often, the thymus of patients and animal models with active autoimmune pathology is burdened by numerous B cell follicles with active germinal centers and plasma cells that are a source of pathogenic autoantibodies.

## Author Contributions

JC, YH, DS, MB, MR, and SN conceptualized, wrote and edited the manuscript. JC and SN designed the figures. All authors contributed to the article and approved the submitted version.

## Funding

This work was supported by FONDECYT 3170424 (SN), FONDECYT 1180385 (DS), FONDECYT 1191438 (MB), CONICYT AFB 170004 (MR), and CONICYT 22201364 (JC).

## Conflict of Interest

The authors declare that the research was conducted in the absence of any commercial or financial relationships that could be construed as a potential conflict of interest.

## Publisher’s Note

All claims expressed in this article are solely those of the authors and do not necessarily represent those of their affiliated organizations, or those of the publisher, the editors and the reviewers. Any product that may be evaluated in this article, or claim that may be made by its manufacturer, is not guaranteed or endorsed by the publisher.
